# Syncope and “Normal” ECG: Is Brugada Syndrome the Culprit?

**DOI:** 10.7759/cureus.82684

**Published:** 2025-04-21

**Authors:** Pamela Ramírez-Rangel, Luis Emilio Bautista-Torres, Miguel Angel Lopez-Lizarraga, Xochitl A Ortiz-Leon, Marco Antonio Ponce-Gallegos

**Affiliations:** 1 Clinical Cardiology, National Institute of Cardiology Ignacio Chávez, Mexico City, MEX; 2 Echocardiography Laboratory, National Institute of Cardiology Ignacio Chávez, Mexico City, MEX

**Keywords:** brugada phenocopies, brugada syndrome, electrocardiogram, syncope, ventricular tachycardia

## Abstract

Brugada syndrome (BrS) is an inherited disorder associated with a risk of ventricular fibrillation (VF) and sudden cardiac death (SCD) in a structurally normal heart mainly in young males, related to pathogenic variants in the *SCN5A* gene. There are three electrocardiographic patterns in BrS that must be identified, being diagnostic only type 1 pattern (coved J-point elevation >2 mm with a negative T-wave in V1 to V3). However, other circumstances can lead to a type 1 Brugada-like ECG, such as atypical right bundle branch block, *pectus excavatum*, arrhythmogenic right ventricular cardiomyopathy, acute coronary syndromes (occlusion of the left anterior descending artery or the conus branch of the right coronary artery), hypokalemia/hyperkalemia and cocaine poisoning, when present, these conditions are known as Brugada phenocopies, and it is imperative to rule out these conditions. We present the case of a young man with syncope with an initial suspected “normal” electrocardiogram with posterior development of type 1 Brugada pattern and an episode of non-sustained ventricular tachycardia.

## Introduction

Brugada syndrome (BrS) was initially identified in 1992 by Pedro and Josep Brugada. The first described case was presented in 1986, corresponding to a three-year-old boy from Poland following multiple episodes of syncope and an abnormal electrocardiogram (ECG) that exhibited ST-segment elevation confined to leads V1 through V3 [1].

This is an inherited condition characterized by an increased risk of sudden cardiac death (SCD), which may be the first manifestation of the disease, despite the absence of structural heart abnormalities. The specific electrocardiographic pattern includes ST-segment elevation and inversion of the T-wave in the right precordial leads (V1-V3) [2].

The underlying mechanisms that cause the characteristic ECG changes and ventricular arrhythmias in BrS remain unclear. Several hypotheses have been suggested, including abnormalities in repolarization, which may lead to the development of lethal arrhythmias. Consequently, the diagnostic approach and accurate risk stratification for SCD are crucial, though challenging [3].

In this report, we present the case of a young man with a history of syncope and dynamic electrocardiographic changes at rest, which ultimately led to the diagnosis of BrS.

## Case presentation

A 35-year-old Latin American man with a history of systemic arterial hypertension treated with losartan twice a day was admitted to the emergency department after experiencing syncope during exercise. A detailed history revealed that the patient also reported having nightmares, and his wife confirmed the presence of nocturnal agonal breathing, although there were no episodes of apnea during sleep. There was no family history of sudden death, and, with respect to his ethnic background, both his parents and grandparents were also Latin American.

**Figure 1:**
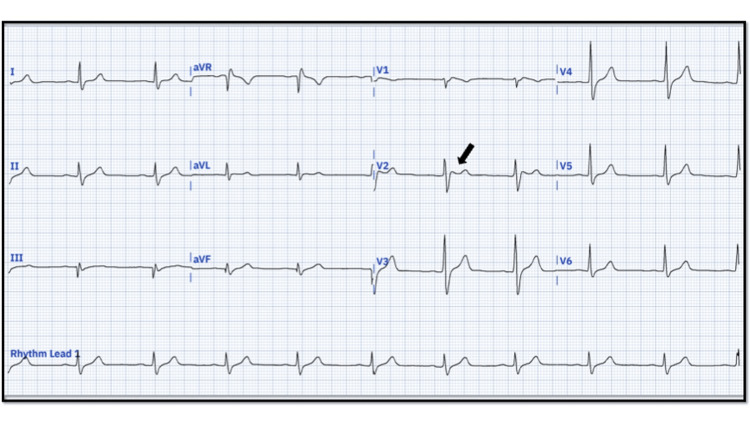
ECG of the patient with type 3 Brugada pattern. The ECG displays a sinus rhythm at 70 beats per minute. aQRS at +30°, PR of 160 ms, and QTm of 412 ms. The saddleback ST-segment layout that makes up the ST-segment elevation can be compatible with a type 3 Brugada pattern (black arrow). Note that in a type 3 Brugada pattern, the ST elevation should be <1 mm in the right precordial lead, followed by a positive T-wave. The owners granted the authors unrestricted access to the most recent version of the PMCardio application (Slovak company Powerful Medical, London).

Physical examination revealed a blood pressure of 145/78 mmHg, a pulse rate of 71 beats per minute, a respiratory rate of 28 breaths per minute, a temperature of 37°C, and an oxygen saturation of 95% on room air. His clinical cardiac evaluation was unremarkable. The ECG at rest (Figure 1) showed a saddleback appearance and <1 mm ST-segment elevation, consistent with a type 3 Brugada pattern. This initial ECG did not exhibit the type 1 BrS pattern.

X-ray radiographs and laboratory values were normal, including troponin, N-terminal pro-B-type natriuretic peptide (NT-proBNP), potassium, and calcium. A transthoracic echocardiogram showed a structurally normal heart with no evidence of hypertensive cardiomyopathy.

**Figure 2:**
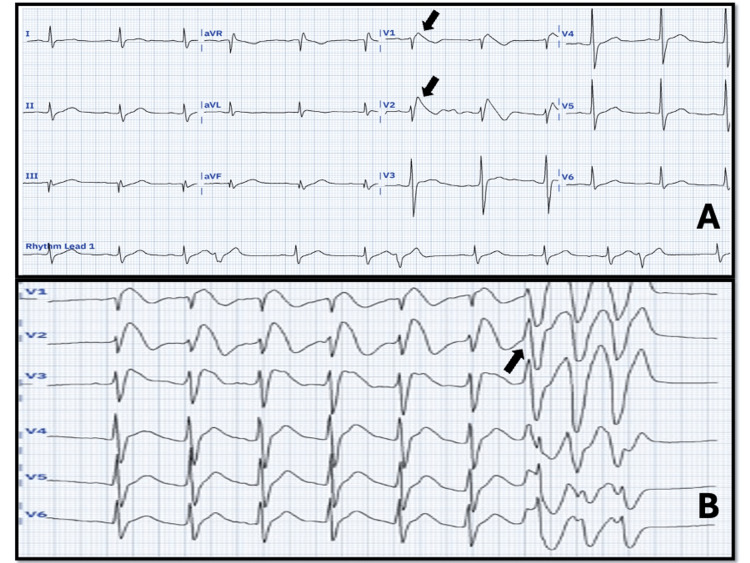
Changes in the ECG of the patient A) ECG shows a sinus rhythm, rate of 66 BPM, with type 1 Brugada pattern which shows concave ST-segment elevation ≥ 2 mm in >1 right precordial lead followed by a negative T-wave (“coved” pattern) (black arrow). B) ECG shows the type 1 Brugada pattern followed by the onset of non-sustained VT (black arrow). The owners granted the authors unrestricted access to the most recent version of the PMCardio application.

Continuous telemetry monitoring (and taking electrocardiograms in resting) in the emergency room revealed dynamic changes in the ST-segment, including coved ST-segment elevation and T-wave inversion, consistent with the spontaneous type 1 Brugada pattern (Figure 2A) and non-sustained ventricular tachycardia (VT) at rest (Figure 2B).

As part of the clinical workup, other causes of ST-segment elevation were ruled out, including acute coronary syndromes, hypokalemia/hyperkalemia, atypical right bundle branch block (RBBB), pectus excavatum, arrhythmogenic right ventricular dysplasia/cardiomyopathy, acute pericarditis, pulmonary embolism, and cocaine poisoning. Placement of venous access, hydration, and monitoring were given in the emergency department. The diagnosis of BrS was confirmed based on the spontaneous Type 1 ECG pattern, along with VT and arrhythmia-related symptoms (syncope and nocturnal agonal respiration). Genetic testing for the *SCN5A* gene was not performed due to its unavailability.

Given the high risk of SCD (based on the presence of VT and syncope), an implantable cardioverter-defibrillator (ICD) was implanted. At the 12-month follow-up, there were no ICD discharges or arrhythmic events. Lifestyle modifications, including avoiding large meals, fever, alcohol, and cocaine consumption, were also recommended.

## Discussion

BrS is an inherited disorder associated with the risk of ventricular fibrillation (VF) and SCD in a structurally normal heart. The global prevalence is 0.05%, but it varies by region, with the highest prevalence found in Southeast Asia, where it reaches 3.7 per 1,000 individuals, predominantly affecting young males. BrS is believed to account for 4% to 12% of all SCDs and as much as 20% of sudden deaths in individuals with structurally normal hearts (1,4). Clinical manifestations of BrS include syncope, seizures, and nocturnal agonal breathing, which are associated with VT or FT [4].

The majority of pathogenic variants identified to date are found in the SCN5A gene, which encodes the alpha-subunit of the Nav1.5 sodium channel. This mutation results in an earlier inactivation and delayed activation of the channel, shortening the duration of the action potential [5,6]. However, this mutation is only present in approximately 30% of patients, suggesting that the disease is genetically heterogeneous.

While the exact pathophysiology of BrS is not fully defined, various experimental studies have provided insights into the mechanisms involved in the two main characteristics of the disorder: the characteristic morphology of the ECG and the predisposition to VT and SCD [4].

The ST-segment changes seen on the ECG are believed to result from differences in the endocardial and epicardial potentials action, particularly due to an increased transient outward potassium current (Ito) in the right ventricular outflow tract (RVOT) epicardium during the early phase of repolarization (phase 2). This causes a local voltage gradient. The Ito current is more prominent in the RVOT, leading to the characteristic ECG changes in the right precordial leads (V1 to V3) [5,7,8].

The predisposition to VT and VF is thought to be caused by regional differences in conduction velocity within the RV epicardium, which can trigger the occurrence of epicardial reentrant excitation waves [5].

**Figure 3:**
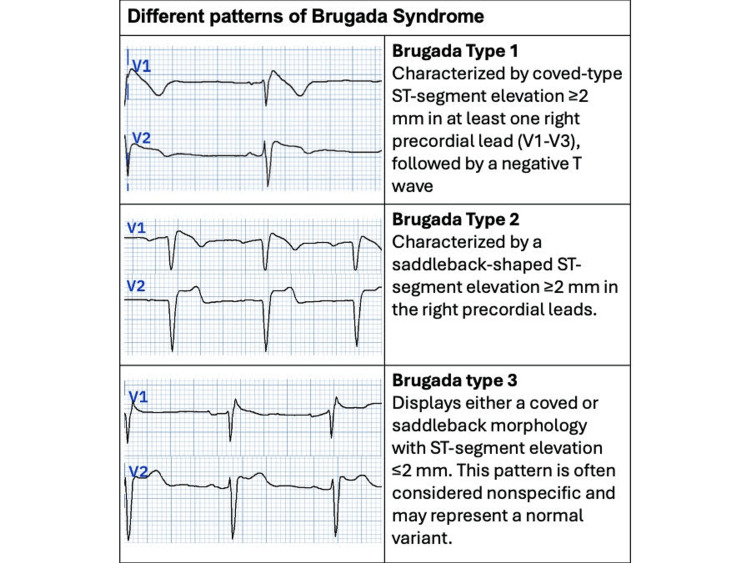
Electrocardiogram patterns of Brugada syndrome Different patterns of Brugada syndrome and their complete description.

There are three ECG pattern types in BrS (Figure 3).

**Figure 4:**
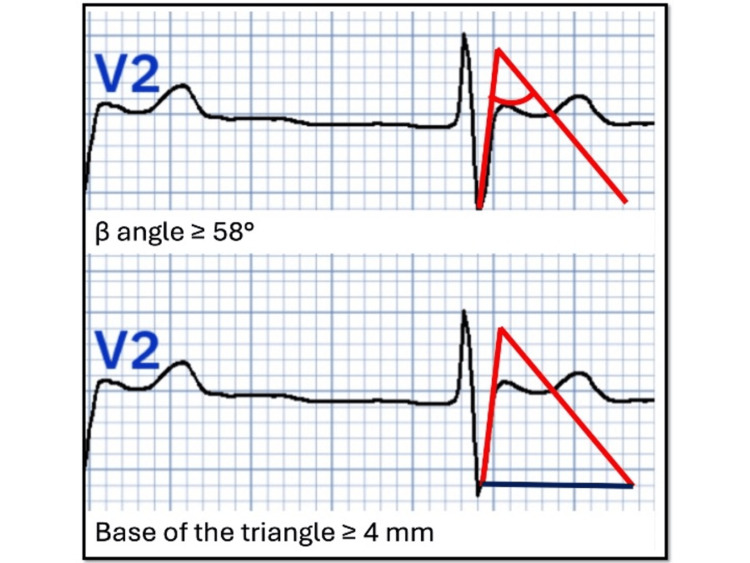
Graphical explanation of the additional criteria for the diagnosis of type 2 Brugada pattern. β angle ≥ 58º and length of the base triangle of the rʹ-wave ≥ 4 mm below the maximum rise point. (The authors were allowed free access to the current version of the PMCardio application by the owners/distributors).

Type 1: Characterized by coved J-point elevation >2 mm and a negative T-wave in the right precordial leads (V1 to V3 in the standard lead position). Type 2: Characterized by ST-segment elevation >0.5 mm in more than one right precordial lead, followed by a convex ST-segment and a positive T-wave in V2. Additional diagnostic criteria, such as a β-angle ≥ 58º and a base length of the rʹ-wave triangle ≥ 4 mm below the point of maximum rise (Figure 4), should be considered to improve diagnostic accuracy, although these criteria are not universally adopted. Type 3: Characterized by a coved or saddleback appearance and <1 mm ST-segment elevation.

It is important to note that the type 1 pattern is the only diagnostic ECG pattern in Brugada syndrome. The type 2 and type 3 Brugada patterns are not diagnostic and require further testing (e.g., pharmacological challenge) or spontaneous progression to the type 1 pattern for confirmation [2,4,9].

A pharmacological test using a sodium-channel blocking agent (such as ajmaline, procainamide, flecainide, or pilsicainide, with a sensitivity of 80%) should be performed when there is a clinical suspicion of BrS (e.g., syncope, agonal respiration, aborted sudden death, or a family history of BrS, or an ECG that is suggestive but not diagnostic), particularly if a spontaneous type 1 ECG pattern is not present. The test aims to unmask the disease [3]. This test must be performed under continuous ECG monitoring and is considered positive when a type 1 ECG pattern is observed during the drug infusion [4,5].

The ECG manifestations of BrS are often dynamic or concealed and may be unmasked or modulated by sodium-channel blockers, a febrile state, vagotonic agents, high vagal tone (large meals, during rest, or while sleeping), α-adrenergic agonists, β-adrenergic blockers, tricyclic or tetracyclic antidepressants, a combination of glucose and insulin, hypo- and hyperkalemia, hypercalcemia, and alcohol and cocaine toxicity. Other variations are frequently seen in the ECG of Brugada syndrome, such as prolonged P-wave, PR interval, or QRS duration, particularly in patients with an SCN5A mutation. The QT interval may occasionally be slightly prolonged in the right precordial leads. Approximately 20% of patients with BrS may develop supraventricular arrhythmias, such as atrial fibrillation [7,8].

As demonstrated in this case, BrS is a dynamic condition, with ECG patterns that can alternate between different types and even include normal recordings in the same patient. This explains why prolonged ECG monitoring has revealed spontaneous, intermittent type 1 ECG patterns in 20% to 34% of patients who only have drug-induced type 1 ECG [4,10].

Respecting gender-specific differences, women with BrS generally exhibit more benign clinical features, including a lower percentage of type 1 BrS ECG patterns and a lower prevalence of symptoms. This is likely due to the influence of hormones and gender-related distribution of ion channels across the heart. Symptoms may be absent or may present as clinical manifestations of arrhythmias, such as syncope, agonal respiration, aborted sudden death, or simply a family history of Brugada syndrome. Men are more likely to be symptomatic than women. The symptoms may be absent or be the clinical expression of the presence of arrhythmias that is, syncope, agonal respiration, aborted sudden death, or only a family history of BrS. Syncope is an indisputable risk factor and is recognized by all studies. The presence of syncope in combination with a spontaneous type 1 ECG pattern is a robust marker of poor prognosis during follow-up. However, it should conduct a thorough evaluation to exclude the possibility of purely vasovagal or neuromodulated syncope, as these patients do not appear to have an increased risk of ventricular arrhythmias during follow-up [4].

Diagnosis on ECG, and risk stratification in BrS patients is challenging, mainly due to the complexity of the condition. Consequently, clinicians fail to offer the appropriate strategy for SCD prevention for many patients. BrS is definitively diagnosed when a type 1 ST-segment elevation is observed in >1 right precordial lead (V1 to V3) in the presence or absence of a sodium-channel-blocking agent, and in conjunction with one of the following [4,5]: 1) Documented ventricular arrhythmias: VF or polymorphic VT; 2) Family history of SCD at <45 years old or coved-type ECGs in family members; 3) Arrhythmia-related symptoms: syncope, seizures, or nocturnal agonal respiration; 4) Inducibility of VT with programmed electrical stimulation.

**Figure 5:**
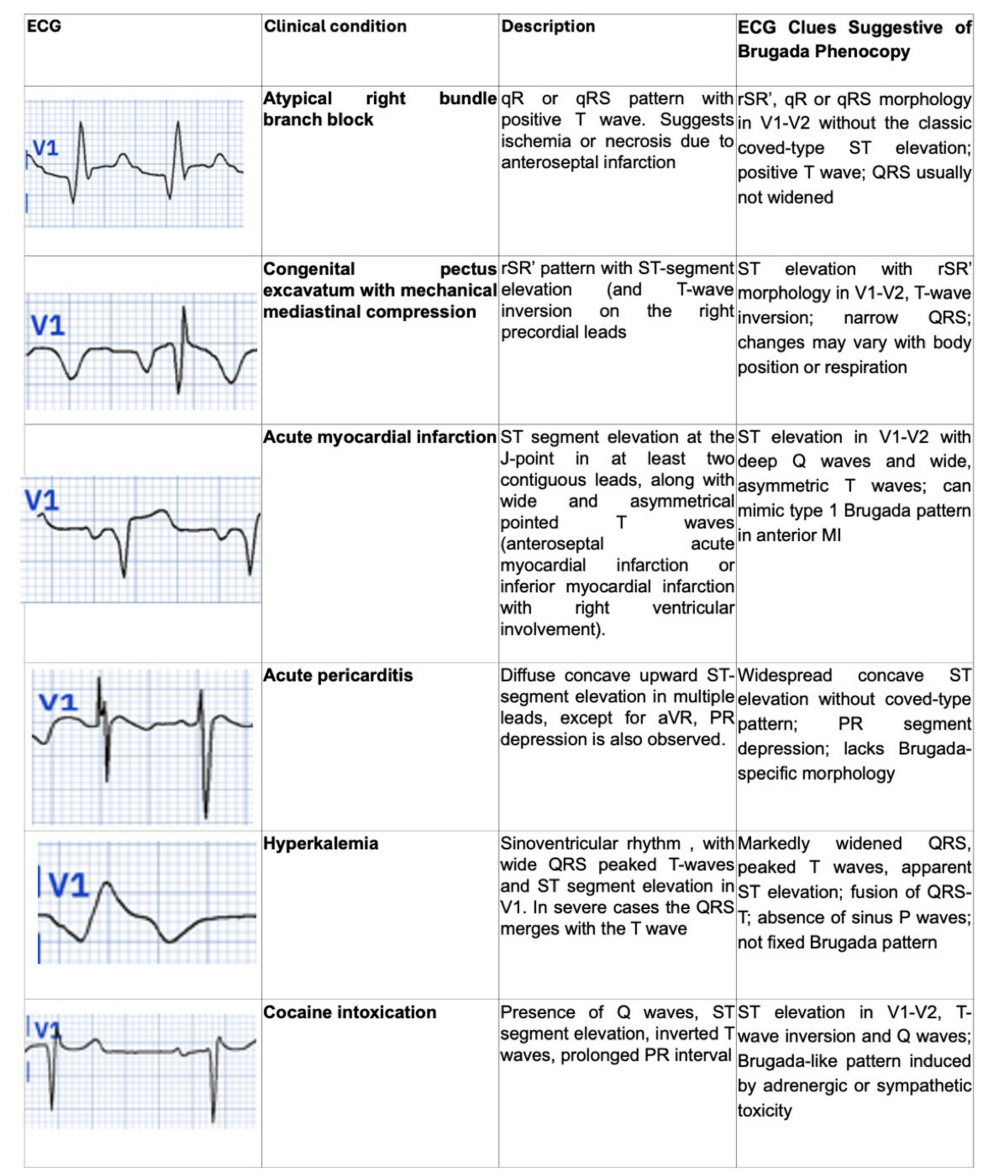
Brugada phenocopies Electrocardiogram example, clinical condition, description, and main clues to differentiate from Brugada syndrome. (The authors were allowed free access to the current version of the PMCardio application by the owners/distributors).

Concerning the differential diagnosis based on ECG, other circumstances can lead to a type 1 Brugada pattern, such as atypical RBBB, pectus excavatum, arrhythmogenic right ventricular dysplasia/cardiomyopathy, acute coronary syndromes (obstruction of the left anterior descending artery or the conus branch of the right coronary artery), hypokalemia/hyperkalemia and cocaine poisoning [4,5,11] (Figure 5).

When these conditions occur, they are termed Brugada phenocopies (BrP). Key diagnostic criteria include [9,11]: an ECG pattern resembling Brugada syndrome (BrS); a clearly identifiable underlying cause; normalization of the ECG after treating the underlying condition; no family history of type 1 BrS or SCD in first-degree relatives under 45 years of age; absence of symptoms (e.g., syncope, seizures, or nocturnal agonal breathing); and a negative sodium-channel blocker challenge test. Although BrP mimics type 1 Brugada ECG patterns, it does not have the same arrhythmic risk. However, cases of phenocopies should still be followed closely if there is any doubt about true BrS [5,8,12].

There are no established definitive guidelines for risk stratification in asymptomatic patients. Current expert consensus statements and recommendations for preventing SCD neither endorse nor discourage the use of electrophysiological studies (EPS) for risk stratification in BrS. They merely suggest that an ICD may be considered in cases where ventricular arrhythmias are inducible [13].

Despite recent progress, genetic screening does not currently impact prognosis or treatment decisions. Instead, its primary role is to confirm the presence of a pathogenic mutation, which may help clarify symptoms in patients with a BrS-like ECG or ambiguous clinical presentations (4,5). Based on current data, clinical guidelines recommend performing a comprehensive genetic analysis specifically for the SCN5A gene [10]. A family history of syncope or SCD - especially involving multiple relatives - along with clinical symptoms, may support a BrS diagnosis [14]. However, these factors alone should not be the only criterion for diagnosis.

The Shanghai Brugada Score System was developed to address the limitations of induced type 1 ECG changes. This scoring system evaluates four key factors: ECG characteristics, clinical history, family history, and genetic testing results. A score >3.5 suggests a probable or definitive diagnosis, 2-3 points indicates a possible diagnosis, and <2 points is considered nondiagnostic [15]. In this case, the patient had a Shanghai Score of 6.5 (despite the unavailability of genetic testing), strongly supporting a definitive/probable BrS diagnosis. However, data on the routine clinical application of this scoring system remains limited. Additionally, studies indicate that the Shanghai Score may fluctuate over time, underscoring the need for regular follow-up and ongoing risk reassessment. Also, clinicians can rely on the Shanghai Score for diagnosis and risk stratification, because evidence has shown that could effectively identify BrS patients at high risk for VF recurrence [15].

Regarding other alternatives for diagnosis, EPS can help in SCD risk assessment by detecting inducible arrhythmias, particularly in asymptomatic patients. Several pharmacological treatments, particularly quinidine and phosphodiesterase inhibitors, are currently used in BrS, though further research is needed to confirm their efficacy. Additionally, radiofrequency ablation of ventricular ectopy has been proposed as a potential therapeutic option.

## Conclusions

BrS remains a diagnostically complex and potentially life-threatening condition, particularly in young individuals with structurally normal hearts. This case illustrates the challenges in identifying BrS, as initial ECGs may appear normal or show only subtle abnormalities, while dynamic changes such as spontaneous type 1 patterns or VT, may emerge later. The presence of syncope, nocturnal agonal breathing, and a high Shanghai Score (6.5) strongly supported the diagnosis, despite the absence of genetic testing or family history of SCD. Further, there are multiple entities that can present as a BrP and must be differentiated from BrS.

Current diagnostic criteria rely heavily on ECG findings, clinical history, and exclusion of phenocopies, yet risk stratification remains imperfect, especially in asymptomatic patients. While ICDs are the mainstay for high-risk individuals, emerging therapies like quinidine and radiofrequency ablation require further validation.

This case underscores the importance of prolonged ECG monitoring in patients with unexplained syncope, even when initial evaluations are unremarkable. Clinicians must maintain a high index of suspicion for BrS, particularly in young males with arrhythmia-related symptoms, to prevent catastrophic outcomes. Future research should focus on refining risk assessment tools, expanding genetic insights, and optimizing therapeutic strategies for this enigmatic syndrome.
